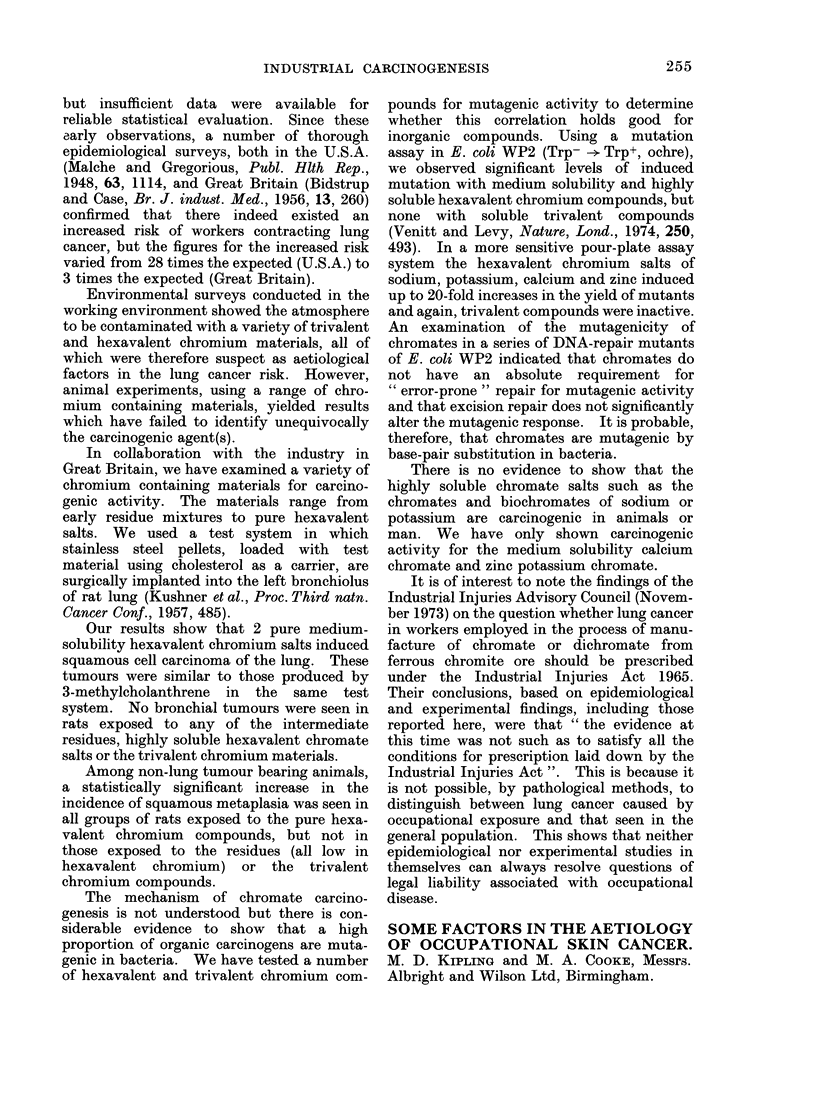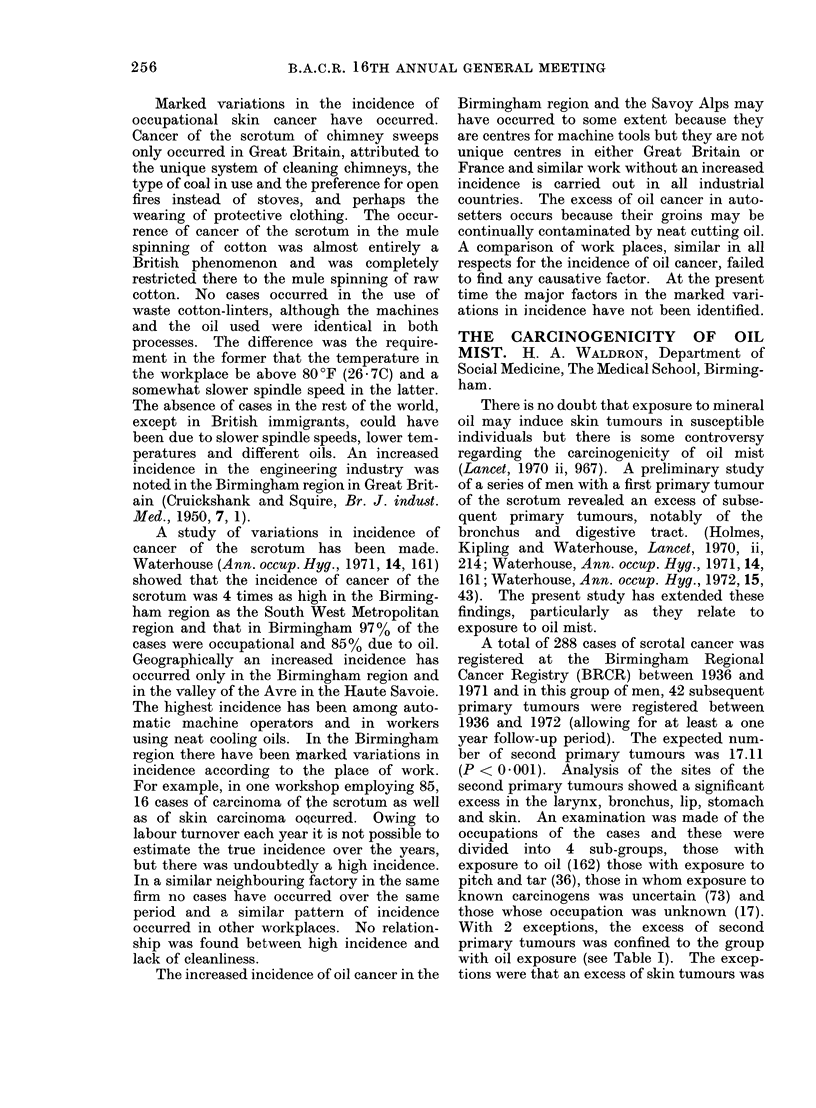# Proceedings: Some factors in the aetiology of occupational skin cancer.

**DOI:** 10.1038/bjc.1975.202

**Published:** 1975-08

**Authors:** M. D. Kipling, M. A. Cooke


### SOME FACTORS IN THE AETIOLOGY OF OCCUPATIONAL SKIN CANCER.

M. D. Kipling and M. A. Cooke, Messrs. Albright and Wilson Ltd, Birmingham.

Marked variations in the incidence of occupational skin cancer have occurred. Cancer of the scrotum of chimney sweeps only occurred in Great Britain, attributed to the unique system of cleaning chimneys, the type of coal in use and the preference for open fires instead of stoves, and perhaps the wearing of protective clothing. The occurrence of cancer of the scrotum in the mule spinning of cotton was almost entirely a British phenomenon and was completely restricted there to the mule spinning of raw cotton. No cases occurred in the use of waste cotton-linters, although the machines and the oil used were identical in both processes. The difference was the requirement in the former that the temperature in the workplace be above 80 °F (26·7C) and a somewhat slower spindle speed in the latter. The absence of cases in the rest of the world, except in British immigrants, could have been due to slower spindle speeds, lower temperatures and different oils. An increased incidence in the engineering industry was noted in the Birmingham region in Great Britain (Cruickshank and Squire, *Br. J. indust. Med.*, 1950, **7**, 1).

A study of variations in incidence of cancer of the scrotum has been made. Waterhouse (*Ann. occup. Hyg.*, 1971, **14**, 161) showed that the incidence of cancer of the scrotum was 4 times as high in the Birmingham region as the South West Metropolitan region and that in Birmingham 97% of the cases were occupational and 85% due to oil. Geographically an increased incidence has occurred only in the Birmingham region and in the valley of the Avre in the Haute Savoie. The highest incidence has been among automatic machine operators and in workers using neat cooling oils. In the Birmingham region there have been marked variations in incidence according to the place of work. For example, in one workshop employing 85, 16 cases of carcinoma of the scrotum as well as of skin carcinoma occurred. Owing to labour turnover each year it is not possible to estimate the true incidence over the years, but there was undoubtedly a high incidence. In a similar neighbouring factory in the same firm no cases have occurred over the same period and a similar pattern of incidence occurred in other workplaces. No relationship was found between high incidence and lack of cleanliness.

The increased incidence of oil cancer in the Birmingham region and the Savoy Alps may have occurred to some extent because they are centres for machine tools but they are not unique centres in either Great Britain or France and similar work without an increased incidence is carried out in all industrial countries. The excess of oil cancer in auto-setters occurs because their groins may be continually contaminated by neat cutting oil. A comparison of work places, similar in all respects for the incidence of oil cancer, failed to find any causative factor. At the present time the major factors in the marked variations in incidence have not been identified.